# Surfing the internet for health information: an italian survey on use and population choices

**DOI:** 10.1186/1472-6947-11-21

**Published:** 2011-04-07

**Authors:** Roberta Siliquini, Michele Ceruti, Emanuela Lovato, Fabrizio Bert, Stefania Bruno, Elisabetta De Vito, Giorgio Liguori, Lamberto Manzoli, Gabriele Messina, Davide Minniti, Giuseppe La Torre

**Affiliations:** 1 Department of Public Health - University of Turin - Turin, Italy; 2 School of Specialization in Public Health - Department of Public Health - University of Turin - Turin, Italy; 3 Institute of Hygiene - University of Sacred Heart of Rome - Rome, Italy; 4 Department Health and Sport Science - University of Cassino - Cassino, Italy; 5 Chair of Epidemiology and Hygiene, Department of Studies of Institutions and Territorial Systems, University "Parthenope" of Naples - Naples, Italy; 6 Section of Epidemiology and Public Health - University "G. D'Annunzio" of Chieti - Chieti, Italy; 7 Department of Public Health, Health Service Research Laboratory - University of Siena - Siena, Italy; 8 Health Service Organization - Rivoli Hospital - Rivoli (Turin), Italy; 9 Department of Public Health and Infectious Diseases -University "Sapienza" of Rome - Rome, Italy

**Keywords:** Cross-sectional, Health, Internet, Behavior

## Abstract

**Background:**

Recent international sources have described how the rapid expansion of the Internet has precipitated an increase in its use by the general population to search for medical information. Most studies on e-health use investigated either through the prevalence of such use and the social and income patterns of users in selected populations, or the psychological consequences and satisfaction experienced by patients with particular diseases. Few studies have been carried out in Europe that have tried to identify the behavioral consequences of Internet use for health-related purposes in the general population.

The aims of this study are to provide information about the prevalence of Internet use for health-related purposes in Italy according to demographic and socio-cultural features, to investigate the impact of the information found on health-related behaviors and choices and to analyze any differences based on health condition, self-rated health and relationships with health professionals and facilities.

**Methods:**

A multicenter survey was designed within six representative Italian cities. Data were collected through a validated questionnaire administered in hospital laboratories by physicians. Respondents were questioned about their generic condition, their use of the Internet and their health behaviors and choices related to Internet use. Data were analyzed using descriptive statistics and logistic regression to assess any differences by socio-demographic and health-related variables.

**Results:**

The sample included 3018 individuals between the ages of 18 and 65 years. Approximately 65% of respondents reported using the Internet, and 57% of them reported using it to search for health-related information. The main reasons for search on the Internet were faster access and a greater amount of information. People using the Internet more for health-related purposes were younger, female and affected by chronic diseases.

**Conclusions:**

A large number of Internet users search for health information and subsequently modify their health behaviors and relationships with their medical providers. This may suggest a strong public health impact with consequences in all European countries, and it would be prudent to plan educational and prevention programs. However, it could be important to investigate the quality of health-related websites to protect and inform users.

## Background

The Internet is a major tool for seeking information for both professional and private reasons and is becoming a challenging tool for health purposes. An increasing number of people worldwide use it to search for all kinds of information, to communicate, to work or to fill their leisure time [1-3]. In Europe, the rate of penetration of Internet usage in the general population is about 58% [2], and recent data show an increase in this rate in Italy up to 52%, thus actually in the mean of European rates [4,5].

In recent years, the use of the Internet for health-related purposes has been growing considerably worldwide, and is therefore an important subject for research [1-3,6-14]. In the United States, studies have found a rate up to 79% of health-related research in Internet users [11]. Several studies have been published in Europe about this subject [1-3,10,11,15,16], although they are not very current, especially if we consider that Internet usage has been consistently increasing [2,3,11,17-20]. In 2005, Andreassen and coll. found a rate of 71% of health-related seeking in the European Internet users [11].

Some authors have studied this phenomenon only among certain categories of Internet users (e.g., in patients with chronic or somatic disease, such as diabetes, hypertension, rheumatic diseases, cancer, AIDS, heart problems, depression) [7,19,21-26]; others have analyzed this topic only for specific types of use (e.g., the utilization of on-line forums) [7,22].

Most published studies, are conducted mainly in Northern European countries [2,7,11,12,21,27], but up to now no studies have been conducted in Italy and there is lack of studies representative of the general population focusing on behavior modifications related to the growing use of the web for health purposes [10,20].

It has already been suggested, indeed, that an unrestrained growth in the use of the Internet might become a source of risk for possible distortions related to a lack of regulation, certification and revision of uncontrolled websites [15,19,28].

The aims of this study are to provide information concerning the behavior of the general population of a European country in terms of the following:

- Internet use related to health and possible determinants; and

- Impact of the information found on health-related behaviors and choices related to the health of users.

## Methods

### Population and setting

A multicenter, population-based, cross-sectional study was carried out in six cities throughout Italy (Torino, Roma, Cassino, Napoli, Siena, Chieti). These cities were selected in an effort to be as representative as possible of the different geographical areas and behaviors.

Using data from the Italian National Statistical Institute (ISTAT) [18,29], we calculated for each center, using EpiInfo [30], an estimation of the number of the interviews necessary in order to get valid data stratified by age and gender. We use the following rates of Internet usage: 64.45% in male and 60.55% in females for the group 18-44 years; 34.23% in males and 19.5% in females for the group 45-65 years. We considered a +/- 20% of Internet usage as "Worst Acceptable" for results.

Unlike other studies, this study excluded people older than 65 years from the sample because Internet usage in Italy among elderly people is very low [18,29]. Demographic data (ISTAT) of our country show indeed that only 6.9% of people aged 65-74 years are using a computer and 5.5% only are using the Internet [29]. Because of the difficulty in finding a number of respondents as high as necessary for the validity of the study in the older age groups, without any sampling errors according to the low rates of internet use, we decided to postpone the argument in eventual future studies.

Participants were voluntarily recruited in blood analysis labs and outpatient diagnostic centers with the aim of enrolling individuals from the general population (i.e., both healthy and unhealthy subjects). Respondents were asked to provide information about their socio-demographic characteristics, including gender, age, marital status, education and employment.

### The questionnaire

Following a careful review of international literature, we decided to conduct the interview using a paper questionnaire administered and compiled by medical doctors, who were trained in an ad hoc manner, by contributing to its formulation and by participating to a training day for the correct compilation of the questionnaire particularly with attention for differences according to age.

The questionnaire was composed of 36 questions that were previously validated by other studies [2,13,17,22,31,32] and was further tested by a pilot study on a sample of 30 individuals who were excluded from the final study, in order to verify the compliance to the interviews and to increase the reliability. Approval by the Ethics Committee of the University "San Giovanni Battista Hospital" of Torino was obtained, and the participants were asked to sign an informed consent form.

The first part of the questionnaire included questions that captured socio-demographic data, drug use, presence of chronic illness in respondents or relatives, reports of personal experience with health care malpractice, and history of hospitalization in the last 12 months [2,16,17,33]. Similarly to Kummerwold, Renahy and Andreassen studies [1,2,11], respondents were asked to indicate their own self-perception of health, that was assessed with a validated numeric related scale (NRS) from 0 to 10; we considered the range 0 to 5 as "poor", 6 to 8 as "moderate" and 9 to 10 as "excellent".

The second part of the questionnaire was related to the use of the Internet for both general purposes and health-related topics. In particular, respondents were asked if, after their search, they made any of the following decisions:

1. 'to choose/change doctor' and/or 'to access a particular hospital or ambulatory clinic', which were categorized in the analysis as "choices in health delivery";

2. 'to start an alternative therapy (such as acupuncture, homeopathy, herbal medicine, etc.)' and/or 'to buy drugs on the web', which were categorized in the analysis as "choices in self-medication";

3. 'to start a therapy not prescribed' and/or 'to change or suspend the therapy recommended by the doctor', which were categorized in the analysis as "negative behaviors";

4. 'to change lifestyle' and/or 'to join preventive programs (i.e., vaccination, screening)', which were categorized in the analysis as "positive behaviors".

### Statistical analysis

Statistical analyses were carried out using STATA 9 [34]. We performed a descriptive analysis of the sample, stratified by gender, and we calculated odds ratios (OR) with 95% confidence intervals (95% CI) and p values to assess the role of socio-demographic factors (i.e., age, gender, education) and health history variables (i.e., chronic disease in the last year) on health-correlated use of the Internet and its effects on population choices.

Differences in proportions between groups were tested using chi-square (χ^2^) tests.

Finally, a multivariate analysis was performed using a logistic regression to assess the influence of socio-demographic variables and health history variables on use of the Internet health-related purposes.

Statistical significance was set at p < .05.

## Results

### Description of the sample

We interviewed 3018 subjects between the ages of 18 and 65 years [35] from October 2009 to May 2010. The sample was composed of 1078 men (35.7%) and 1924 women (63.7%); the mean age of respondents was 48.8 years (standard deviation [SD] 12.39), with no significant differences by gender (male 48.1 years and female 49.2 years) (data not shown). We found a refusal rate of about 20%, ranking from 15% to 24%.

Internet users were found to be 64.6% of the total sample (N = 1949) (72.1% of males and 60.3% of women) (χ^2 ^= 49.9; p < .001). Twenty-two subjects reported delegating the search of health information, and they were not considered in the analysis. Rate of e-health use (use of the Internet for any health-related purposes) was 56.5% of Internet users, with a significant difference noted between males and females (61.6% of female users and 50.2% of male users; p < .001) (Table 1).

**Table 1:** Description of Internet users (e-health and non e-health users) according to socio-demographic variables (N = 1927)

		E-health users % (N)		Non e-health users % (N)	
		**Male**	**Female**	**Male**	**Female**

Age group	18 -29	60.7(68)	65.5(131)	39.3 (44)	34 (68)

	30-41	61 (94)	78.8 (145)	35.7 (55)	20.7 (38)

	42-53	47.1 (137)	59.0 (271)	51.5(150)	40.1 (184)

	54-65	41 (87)	52.9 (166)	56.6 (120)	46.5(146)

					

	*P value, male*	*<.001*			

	*P value, female*	*<.001*			

					

Marital status	Unmarried	54.9 (147)	65.3 (241)	43.3 (116)	34.4 (127)

	Married	50.4 (198)	61.8 (385)	49.3 (194)	39.2 (244)

	Widower	17.5 (7)	40.6 (13)	82.5 (33)	59.4 (19)

	Separated/Divorced	55.2 (32)	61.9 (73)	43.1 (25)	37.3 (44)

					

	*P value, male*	*.021*			

	*P value, female*	*.328*			

					

Education	Illiterate	0 (0)	0 (0)	0 (0)	100 (1)

	Primary	50.0 (5)	46.7 (7)	50.0 (5)	53.3 (8)

	Middle school	31.6 (36)	55.8 (63)	65.8 (75)	43.4 (49)

	High school	50.8 (210)	63.6 (7)	47.5 (196)	27.3 (3)

	College graduate	58.2 (135)	58.8 (351)	40.1 (93)	40.4 (241)

					

	*P value, male*	*.0057*			

	*P value, female*	*.24*			

					

Work status	Employed	49.6 (314)	61.5 (531)	48.8 (309)	37.8 (327)

	Unemployed	27.8 (5)	63.0 (17)	66.7 (12)	33.3 (9)

	Student	67.3 (37)	70.7 (87)	30.9 (17)	29.3 (36)

	Retired	45.8 (27)	44.0 (29)	50.8 (30)	53.8 (35)

	Housewife	---	60.3 (44)	---	39.7 (29)

					

	*P value, male*	*.28*			

	*P value, female*	*.02*			

Totals may not always be the same because of missing values

Among males, the youngest age group (18-29 year-olds) used the Internet the most for health-related purposes, and e-health use decreased with increasing age in males. However, among females, the highest rate of e-health use was among 30-41 year-olds (78.8%). This age group also displayed the largest gender-related difference in e-health use (78.8% in women versus 61.0% in men; p < .001).

People living alone (i.e., unmarried, separated or divorced) used more e-health than their cohabiting counterparts. Among men, only the difference between the married and widowed groups reached statistical significance (p = .021).

Among men, a higher educational level was associated with a significantly higher rate of Internet use for health-related purposes, even if primary school graduates are well-represented (p = .006). Among women, no significant association with educational level was found (p = .24).

In terms of work status among males, students used the Internet more for health purposes (67.3%), but no significant differences by employment status were found in males (p = .28). It is worth noting that unemployed males reported the lowest rate of e-health use (27.8%).

In women, we found a significant association between employment status and e-health use (p = .02). Also in females, the student group reported the highest rate of e-health use (70.7%), and retired women used the Internet for health purposes less than the other groups (44%).

In contrast with men, unemployed women and students reported using e-health more than other women (63% and 70%, respectively).

Concerning variables related to health status (Table 2), the presence of chronic disease or daily drug use was significantly associated with the use of e-health in both women (p = .01) and men (p = .05).

**Table 2:** Description of Internet users (e-health and non e-health users) by health-related variables (N = 1927)

		E-health users % (N)		Non e-health users % (N)	
		**Male**	**Female**	**Male**	**Female**

Chronic disease	Yes	57.7 (97)	75.6 (242)	38.1 (64)	23.8 (76)

	No	47.8 (286)	56.1 (467)	51 (305)	43.1 (359)

					

	*P value. male*	.0059			

	*P value. female*	<.001			

					

Hospitalization	Yes	52.5 (62)	66.1 (111)	44.9 (53)	32.7 (55)

	No	50.1 (320)	60.8 (598)	48.2 (308)	38.6 (380)

					

	*P value. male*	.8220			

	*P value. female*	.5602			

					

Drug use	Daily	54.2 (83)	73.1 (220)	41.2 (63)	25.9 (78)

	2-3 times a week	34.6 (47)	51.1 (94)	64 (87)	48.4 (89)

	Rarely	51.4 (186)	60.5 (317)	47.5 (172)	39.1 (205)

	Never	58.6 (68)	55.5 (81)	40.5 (47)	443.2 (63)

					

	*P value. male*	.0022			

	*P value. female*	<.001			

					

Health perception	Poor	58 (29)	72.7 (88)	36 (18)	26.4 (32)

	Moderate	49.2 (277)	59.4 (506)	49.2 (277)	39.8 (339)

	Excellent	51.3 (80)	64.7 (119)	47.4 (74)	35.3 (65)

					

	*P value. male*	.2227			

	*P value. female*	.1296			

					

History of Malpractice	Yes	56.6 (125)	68.1 (280)	24.4 (90)	29.4 (128)

	No	47.1 (255)	57.8 (428)	75.3 (278)	70.6 (308)

					

	*P value. male*	.041			

	*P value. female*	.004			

Totals may not always be the same because of missing values

No significant results were found regarding hospitalization or self-perception of health status.

Among subjects reporting personal experience with medical malpractice, the rate of e-health use was 56.6% in males and 68.1% in females, while in the absence of such a personal experience, 47.1% of males and 57.8% of females reported using e-health (p = .004). (Table 2)

### Modification of behaviors

Any health-related modification of behavior was investigated according to socio-demographic and health-related variables (Table 3).

**Table 3:** Association between behavior modifications and e-health use (N = 1107)

			OR	IC 95%	P
Choices in the provision of health	Age	18 -29	1*	---	---

		30-41	0.97	0.66-1.43	.879

		42-53	1.16	0.81-1.65	.421

		54-65	1.17	0.79-1.74	.423

	Gender	Male	1*	---	---

		Female	1.20	0.92-1.55	.171

	Chronic disease	Yes	1*	---	---

		No	0.89	0.68-1.71	.402

	Education	Primary	1*	---	---

		Middle school	1.30	0.35-4.79	.692

		High school	1.02	0.30-3.54	.970

		College graduate	1.01	0.29-3.49	.991

					

Choices in self-medication	Age	18 -29	1*	---	---

		30-41	0.72	0.41-1.28	.265

		42-53	0.58	0.34-0.99	.044

		54-65	0.66	0.37-1.19	.168

	Gender	Male	1*	---	---

		Female	0.69	0.46-1.02	.062

	Chronic disease	Yes	1*	---	---

		No	0.65	0.44-0.97	.035

	Education	Primary	1*	---	---

		Middle school	0.53	0.10-2.80	.454

		High school	0.54	0.11-2.56	.439

		College graduate	0.48	0.10-2.29	.355

					

Negative behaviors	Age	18 -29	1*	---	---

		30-41	1.78	0.95-3.34	.073

		42-53	1.79	1.00-3.22	.049

		54-65	0.96	0.48-1.93	.917

	Gender	Male	1*	---	---

		Female	0.96	0.65-1.41	.824

	Chronic disease	Yes	1*	---	---

		No	0.67	0.46-0.99	.004

	Education	Primary	1*	---	---

		Middle school	1.31	0.15-11.24	.806

		High school	0.96	0.12-7.63	.967

		College graduate	1.71	0.22-13.63	.611

					

Positive behaviors	Age	18 -29	1*	---	---

		30-41	0.93	0.63-1.35	.686

		42-53	0.90	0.64-1.27	.557

		54-65	1.29	0.88-1.89	.192

	Gender	Male	1*	---	---

		Female	1.08	0.84-1.39	.537

	Chronic disease	Yes	1*	---	---

		No	1.17	0.9-1.51	.237

	Education	Primary	1*	---	---

		Middle school	3.14	0.86-11.50	.084

		High school	2.23	0.64-7.70	.206

		College graduate	2.15	0.62-7.46	.227

The most relevant and significant results were found in the "self-medication" and "negative behaviors" categories. The modifications in "choices in the provision of health" and "positive behaviors" were not correlated with e-health use.

There was an association between age and chronic disease with choices in self-medication. Age greater than 30 years had an inverse relationship with self-medication, although this result was significant only for the 42-53 year age group. Being healthy (i.e., not affected by chronic diseases) reduced the probability of engaging in self-medication. Though the differences were not statistically significant, female gender and higher levels of education were associated with lower levels of self-medicating behaviors.

A significantly higher risk of negative behaviors was found with increasing age up to 53 years (p = .05), while the absence of chronic diseases decreased the risk of negative behaviors (p = .004).

### Multivariate analysis

Multivariate analysis (Table 4) revealed that the variables associated with a higher probability of e-health use were the following:

**Table 4:** Results of the multivariate analysis evaluating potential predictors of e-health use

		OR	IC 95%	P value
Gender	Male	1*	-	-

	Female	1.40	1.15-1.70	.001

				

Age		0.55	0.46-0.66	<.001

				

Education	Primary/Middle school	1*	-	-

	High school	1.54	1.14-2.07	<.001

	College graduate	2.32	1.69-3.19	<.001

				

Marital status	Unmarried	1*	-	-

	Married	1.17	0.91-1.50	.21

	Widower	0.50	0.27-0.90	.02

	Separate/Divorce	1.42	0.97-2.08	.07

				

Health perception	Poor	1*	-	-

	Moderate	0.75	0.33-1.68	.48

	Excellent	0.66	0.30-1.48	.31

				

Chronic disease	No	1*	-	-

	Yes	2.56	2.00-3.27	<.001

- female gender (p = .001);

- younger age: considering age as a continuous variable, e-health use decreased with age (OR = 0.55);

- presence of chronic diseases: a significant association was found between e-health use and the presence of a chronic illness (OR = 2.56);

- level of education: e-health use increases with the level of education (p = .001) Self-perception of moderate or excellent health seemed to be associated with a minor use of e-health, although these results were not statistically significant.

We did not find significant differences in e-health use by marital status, except widowers (p = .02) showing a lower use of the e-health.

## Discussion

In recent years, the number of Internet users has dramatically increased worldwide [1-3,5,9,11-13,15,19,27,36,37], and ISTAT data reveal that this phenomenon has occurred in Italy too [4]. As the mean of European rate of Internet use is about 58%, Italy can be considered as representative of Europe [4,5].

The use of the Internet for health-related purposes is also increasing [1-3,6-14], and it is important for public health professionals to observe the trend and consider the possible consequences of such use in the general population.

The aims of this survey were to assess the actual rate of Internet use for health-related purposes, to describe the variables associated with its use and to assess the possible behavioral consequences.

Presently, several studies were published in Europe about this subject [1-3,10,11,15], but only certain categories of Internet users or types of use were evaluated [7,22].

In this survey, we chose not to administer the survey by phone to reduce the likelihood of finding fewer significant results due to higher rates of refusal or incompleteness [38,39]. The decision not to administer the questionnaire online was made to avoid the selection of people using the Internet habitually and with greater skills. Moreover, this study may be innovative for the choice of conducting face-to-face interviews.

To get a representative sample of the general population, we performed the study in different Italian regions in both urban and rural contexts, and interviews were carried out in both blood analysis labs and outpatient diagnostic centers. However, this may be a limitation of this study due to geographical differences in the way people access and interface with health services based on the social and cultural patterns of the geographical center. Moreover, people accessing blood labs or diagnostic centers may be more involved with their own health or more apt to spend time answering health-related questions than the average population.

Another limitation may be the personal predisposition to accept the interview: for example, people refusing to answer the questions may be introspective or shy and therefore a potential user of an online forum and a seeker of information on the Internet. This represents the possible loss of a potentially important part of the sample. Thus, we tried to overcome this problem by clearly specifying the objectives of the study at the proposal of the interview. Moreover, the decision to use physicians to administer the questionnaire enabled us to gain greater participation in the study and to achieve better compliance and completeness of the questionnaires, obtaining thus an essentially low refusal rate.

In our findings, the Italian rate of Internet use for health-related purposes is about 56.5% of Internet users, a result similar to what found in other European countries [2,7,11,17,33], thus suggesting that the use of technologies for health is almost similar among European Internet users.

Internet use for health-related purposes was higher among women, even though men were using the Internet more for all purposes. This finding is consistent with previous European studies [10,17,36,40].

The variables more correlated with both e-health use and a higher risk of possible wrong use were female gender, presence of chronic disease and younger age. Women in particular seemed to be more influenced by e-health than men in terms of subsequent health choices.

Elderly Internet users were less likely than younger users to access the web for health-related purposes. In our study, Internet users were properly represented in all age groups; therefore these results should be interpreted as a consequence of a lower propensity among the elderly towards the use of the Internet for health-related purposes, rather than a reflection of their general Internet use patterns.

Individuals with a low-middle school level of education reported the lowest rate of e-health use. This could be explained by a possible effect of work status: people with a low-middle school level of education are more likely to work in manual labor, and thus they are likely to be less concerned with technology and the Internet.

As in the European study carried out by Andreassen [11], we did not find statistically valid results about the relationship between self-perception of health and e-health use, but as in other European studies, a trend of opposite correlation was found between the subjective assessment of health status and the use of the e-health [1,7,11,17,33]. A larger sample is probably necessary for a better analysis of self-perception of health in Internet users.

Multivariate data analysis also showed an association between health status and e-health seeking. The presence of any chronic disease tended to facilitate these behaviors, suggesting that the Internet seemed to be an important information-seeking tool for people affected by a chronic illness. These results are in accordance with the findings of previous studies, even those that focused only on populations with specific diseases [1,2,11,22,37,40].

The rate of users adopting any dangerous modifications of behavior based on information found on the Internet was quite high and noteworthy, especially in those with chronic diseases. These results are very important because such behaviors may be dangerous, especially if they are executed on the basis of false information.

As confirmed by this study, there are a considerable number of people seeking health information on the Internet and changing their behavior and choices based on the information that they find. Users may misunderstand information found online, and they may overestimate their knowledge and their ability to judge what they find.

Wrong choices and hazardous behaviors may indeed result from poor quality and reliability of information; therefore, control of information is very important. As other authors have suggested [41-43], it may be important to investigate the accessibility of institutional websites and evaluate their accuracy and appropriateness.

Future studies are needed to investigate the quality of health information found on the web, especially for disease information or medical questions that are frequent search topics and are more correlated with behaviors and choices.

## Conclusions

The Internet may be considered a useful tool, even for health care providers, to promote educational initiatives on behaviors, lifestyles, and screening programs [17,44].

An important function of public health may be to promote the correct use of e-health (especially in trigger groups identified in this study) to reduce hazardous behaviors.

An additional goal may be to develop a higher sensitization to e-health use in the general population (i.e., both healthy and unhealthy individuals) for the endorsement of large-scale online prevention and lifestyle education programs.

## Competing interests

The authors declare that they have no competing interests.

## Authors' contributions

All Authors have seen and approved the submitted manuscript. RS participated in the study design, interpreted the results and revised the manuscript; MC and EL participated in acquiring the data, analyses performing and drafting the paper; FB participated in interpreting the results and revising the manuscript; SB, EDV, GL, LM, DM and GM participated in acquiring the data and interpreting the results; GLT participated in acquiring the data, interpreting the results and revising the manuscript.

## Pre-publication history

The pre-publication history for this paper can be accessed here:

http://www.biomedcentral.com/1472-6947/11/21/prepub
